# Primer-Less Species Identification Throughout Fungal (*Tuber magnatum*), Plant (*Corylus avellana*) and Animal (*Eisenia fetida*) Kingdoms by Direct RNA Sequencing

**DOI:** 10.3390/ijms27156549

**Published:** 2026-07-23

**Authors:** Tadeusz Malewski, Slavica Matić, Aleksandra Gabriela Bilska, Maria Alexandra Cucu, Laura Miozzi, Antonietta Mello, Andrzej Skwiercz, Tomasz Oszako, Justyna Anna Nowakowska

**Affiliations:** 1 Department of Molecular Evolution, Museum and Institute of Zoology, Polish Academy of Sciences, 00-818 Warsaw, Poland; abilska@miiz.waw.pl; 2 Institute for Sustainable Plant Protection (IPSP), National Research Council of Italy (CNR), Strada delle Cacce 73, 10135 Turin, Italy; slavica.matic@unipa.it (S.M.); mariaalexandra.cucu@crea.gov.it (M.A.C.); laura.miozzi@cnr.it (L.M.); 3 Department of Agricultural, Food and Forest Sciences (SAAF), University of Palermo, Viale delle Scienze, 90128 Palermo, Italy; 4 Doctoral School of Molecular Medicine, Medical University of Lodz, 90-419 Lodz, Poland; 5 Council for Agricultural Research and Economics—Research Centre for Plant Protection and Certification (CREA-DC), Via Di Lanciola 12A, 50125 Firenze, Italy; 6 Institute for Sustainable Plant Protection (IPSP), National Research Council of Italy (CNR), Viale Pier Andrea Mattioli 25, 10125 Turin, Italy; antonietta.mello@cnr.it; 7 The National Institute of Horticultural Research, 96-100 Skierniewice, Poland; andrzej.skwiercz@inhort.pl; 8 Forest Protection Department, Forest Research Institute, 05-090 Raszyn, Poland; t.oszako@ibles.waw.pl; 9 Faculty of Biology and Environmental Sciences, Institute of Biological Sciences, Cardinal Stefan Wyszyński University in Warsaw, 01-938 Warsaw, Poland; j.nowakowska@uksw.edu.pl

**Keywords:** species identification, primer-free barcoding, direct RNA sequencing, biodiversity assessment

## Abstract

Accurate species identification is essential for biodiversity studies, ecological monitoring, and biosecurity, but current molecular approaches rely on PCR amplification, which requires universal primers. Here, we evaluated the possibility of primer-free species identification via direct RNA sequencing (dRNA-seq) by using Oxford Nanopore Technology (ONT). Total RNA isolated from three major eukaryotic kingdoms—fungal (*Tuber magnatum* Picco 1788), plant (*Corylus avellana* L. 1753), and animal (*Eisenia fetida* Savigny 1826) specimens—was sequenced without reverse transcription and PCR amplification. Taxonomic assignments based on similarity to ribosomal nuclear (fungi and animals) and chloroplast (plant) transcripts enabled reliable host species identification. Ribosomal RNA reads dominated the datasets and supported accurate identification of the target organisms, while additional sequences revealed associated microbiota and co-occurring taxa. Notably, dRNA-seq successfully detected symbiotic bacteria in *E. fetida* and latent fungal infection in *C. avellana*, highlighting the method sensitivity. However, the complex organization of nuclear ribosomal gene clusters may complicate taxonomic assignment in plants, underscoring the need for improved analytical pipelines. Overall, our results provide proof of concept that dRNA-seq enables primer-independent species identification while simultaneously providing insights into the host microbiomes. Direct sequencing of naturally present RNA molecules generated sufficient sequence information for species identification across different taxonomic kingdoms, indicating the technical feasibility of the developed technique. Further validation using larger biological datasets and direct comparisons with conventional sequencing methods will determine its wider applicability as an innovative and complementary species identification approach.

## 1. Introduction

Accurate biological classification is fundamental to biodiversity studies, environmental surveillance, invasive species prevention, and wildlife conservation. Although traditional taxonomy relies on morphological traits, its accuracy can be limited by phenotypic plasticity, cryptic species, and the need for specialized taxonomic expertise. Over the past two decades, molecular approaches, most notably DNA barcoding, have transformed species identification by providing standardized genetic markers for taxonomic assignment [1]. By using short, standardized gene regions with sufficient interspecific variability, DNA barcoding enables species discrimination, while conserved flanking sequences facilitate primer design across diverse taxa [2,3]. Consequently, this approach has proven to be a reliable, cost-effective, and high-throughput tool for species identification [4,5].

The emergence of high-throughput sequencing (HTS) technologies has further expanded the scope of molecular identification by enabling simultaneous detection of multiple species within complex samples. Metabarcoding approaches currently enable the comprehensive characterization of biological communities from environmental DNA (eDNA), facilitating ecological investigations across diverse ecosystems and taxonomic groups [5]. These methods have been successfully applied to bacteria, protists, fungi, plants, and animals, offering new insights into community composition and ecosystem functioning [6,7,8].

Despite these advances, both barcoding and metabarcoding depend critically on the availability and efficiency of universal primers. Designing primers that balance broad taxonomic coverage with sufficient discriminatory power remains a major challenge. In animals, commonly used mitochondrial COI primers fail to amplify certain taxa, including nematodes [9,10,11], sponges [12,13], and cnidarians [14,15]. In fungi, the internal transcribed spacer (ITS) region is widely used but exhibits variable amplification success across lineages, from 100% in Saccharomycotina and Basidiomycota to 65% in Mucoromycotina, Chytridiomycota, and Blastocladiomycotina [16,17]. In plants, no single universal barcode has been universally accepted, and multilocus barcoding approaches are increasingly adopted [18,19]. Similar limitations exist in protists [20,21] and myxomycetes [22], where extensive diversity complicates the design of broadly effective primers.

Third-generation sequencing technologies have significantly expanded the possibilities of studying biodiversity at the molecular level. Among third-generation technologies, the platform developed by Oxford Nanopore Technology (ONT) is unique, capable of sequencing RNA directly, rather than relying on reverse transcription and PCR, and is suitable for direct field application [23,24,25]. In contrast to short-read sequencing technologies, nanopore sequencing generates long reads capable of spanning entire ribosomal transcription units, thereby retaining phylogenetically informative regions within a single read [26,27]. Furthermore, the high abundance of ribosomal RNA reduces sequencing depth requirements. This approach provides primer-free sequencing of rRNA and other phylogenetically informative markers [28,29], offering new opportunities for species identification. Together with its portability and cost-effectiveness, these characteristics make ONT direct RNA sequencing an attractive tool for species identification, biodiversity studies, and molecular diagnostics.

**Figure 1:**
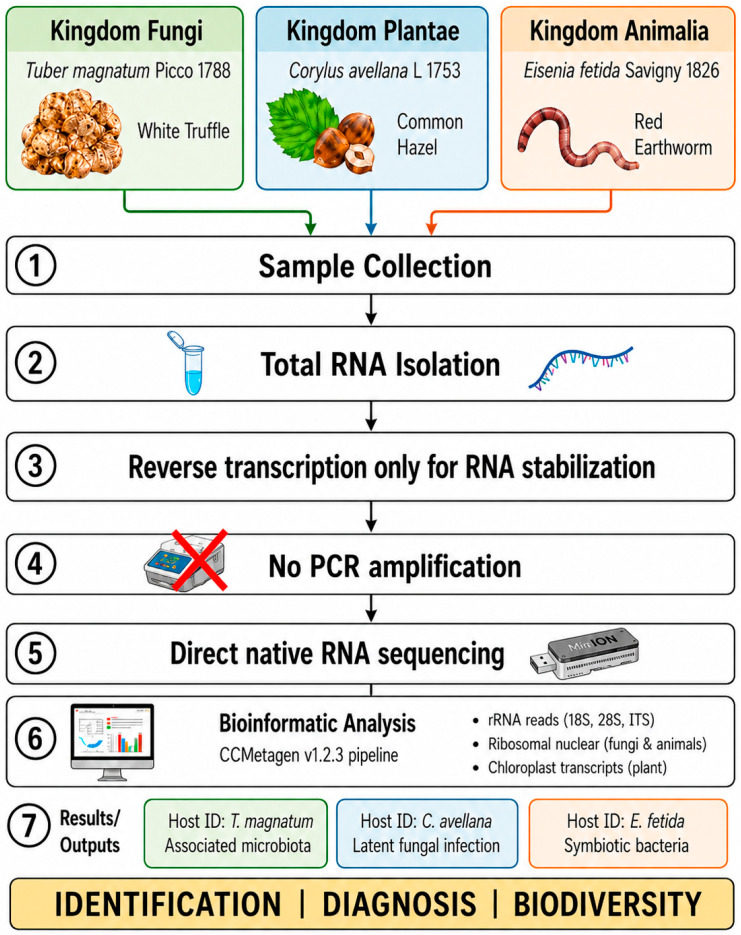
Workflow of primer-free species identification by direct RNA sequencing.

This proof-of-concept study assessed the technical feasibility of direct RNA sequencing (dRNA-seq) as a primer-less strategy for species identification across different taxonomic units (Figure 1). Rather than comparing its analytical performance with other conventional DNA barcoding methods, the objective of our study was to evaluate whether naturally present RNA molecules could be used directly for the identification of taxonomically different species and their associated microbiota using a primer-free dRNA-seq technique. We hypothesized that native RNA molecules would be sufficiently abundant to enable specific, direct species identification originating from different taxonomic kingdoms. Our results provide initial insight into how dRNA-seq can identify species without the use of universal primers and offer additional biological information beyond traditional DNA barcoding. To our knowledge, this is the first study to evaluate the use of the dRNA-seq method across three major eukaryotic kingdoms, allowing accurate species identification of the host alongside its associated microbiomes.

## 2. Results

### 2.1. Sequencing Output and Read Quality

Total RNA isolated from fungal (*Tuber magnatum*), plant (*Corylus avellana*), and animal (*Eisenia fetida*) specimens was analyzed using nanopore dRNA-seq on the MinION platform. Six-hour sequencing runs generated approximately 165,000, 132,000, and 148,000 reads for the fungal, plant, and animal samples, respectively. Median read lengths ranged from 846 to 982 nt, with median quality scores between 9.8 and 10.1.

**Figure 2:**
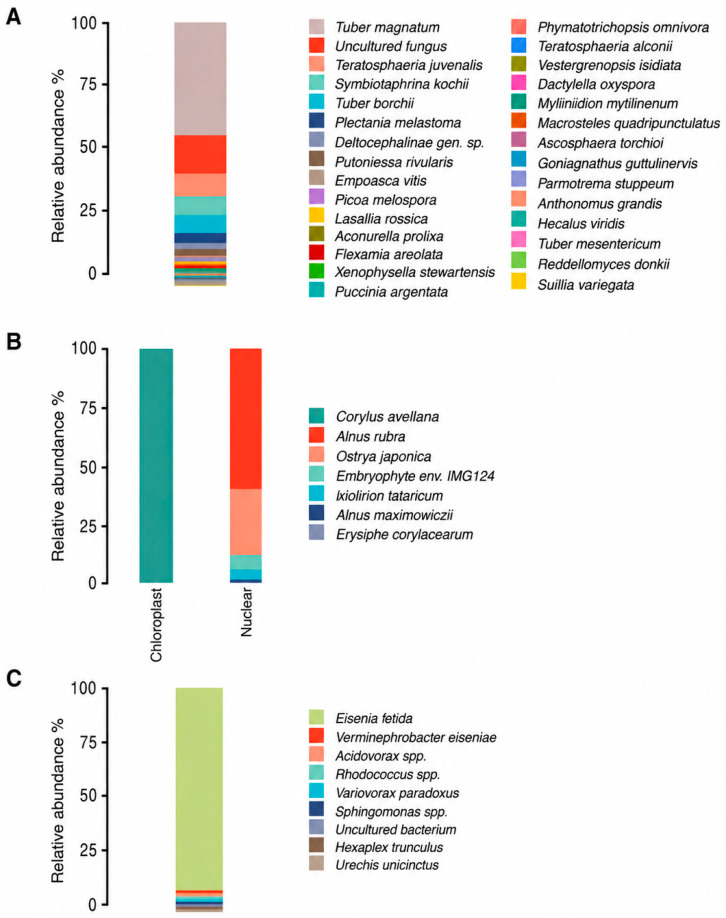
Relative species abundance detected in the *Tuber magnatum* sporocarp (**A**), *Corylus avellana* leaf (**B**), and *Eisenia fetida* (**C**) samples. The rest of the reads were identified at other taxonomical levels.

Ribosomal RNA sequences dominated all datasets, consistent with the natural composition of total cellular RNA, in which rRNA typically accounts for more than 80% of transcripts. The high abundance of rRNA molecules, together with the ability of direct RNA sequencing to generate long reads spanning multiple variable regions, makes rRNA a suitable target for taxonomic identification. The relative abundance of host-derived reads and associated non-host taxa was summarized for all three samples (Figure 2).

### 2.2. Fungal Sample: Tuber magnatum

Taxonomic classification assigned 72.4% of reads to the species, 20.4% to the genus level, and the remaining 7.2% to higher taxonomic ranks. Most reads were classified as fungal (84.5%), and the remaining 7.0% mapped to other eukaryotes or remained unclassified (Figure 2A).

The largest fraction of reads were assigned to *T. magnatum*, including 26.4% matching 18S rRNA sequences and 6.5% matching the ITS1–5.8S–ITS2 region, resulting in a combined abundance of 32.9%. An additional 17.7% of reads were assigned to *Tuber* spp. based on matches with 28S rRNA sequences (GenBank KY427072.1 and JX087962.1), while 7.3% of reads were attributed to other species within the genus. The obtained results support the correct taxonomic identification of the analyzed sporocarp as *T. magnatum*.

Although the primary objective of this study was to use dRNA-seq for species identification, the obtained data also provided insights into the associated microbial communities. The detected taxa span diverse ecological groups, including co-occurring hypogeous genera (*Picoa*, *Reddellomyces*), soil saprotrophs (*Dactylella*, *Plectania*), and insect-associated symbionts (*Ascosphaera*, *Symbiotaphrina*). Additionally, sequences from lichenized and epiphytic fungi reflect environmental input from the nearby plant canopy.

Taxonomic analysis of the non-fungal eukaryotic reads showed a predominance of insect sequences, primarily belonging to the infraorder Cicadomorpha (leafhoppers). Among the insect taxa detected, the most abundant were phytophagous, including *Deltocephalinae* gen. sp. (1.7%), *Putoniessa* (1.6%), and *Empoasca vitis* (1.1%). Because these insects reside on above-ground vegetation, their relatively high abundance may suggest a significant environmental DNA drift, where canopy biomass (e.g., exuviae, honeydew) accumulates on the forest floor and adheres to the truffle peridium.

At the same time, the dataset captured signs of belowground biotic interactions. Some detected reads were assigned to the truffle fly *Suillia variegata* (0.1%), a specialized dipteran that lays eggs directly on mature, underground fruiting bodies. The simultaneous detection of distinct sequences from beetles (*Anthonomus grandis*—0.2%; *Melyridae* sp.—0.1%) further suggests the use of the truffle matrix by macroinvertebrates, demonstrating that ONT profiling of sporocarps effectively captures both transient environmental background noise and close ecological connections.

In addition to the dominant sequence reads from fungi and insects, dRNA-seq analysis identified soil bacteria, plants, and microscopic eukaryotes. Rhizosphere bacteria, including *Pedobacter steynii* and *Pseudorhodoferax soli*, which could potentially contribute to soil organic matter decomposition, were also detected.

### 2.3. Plant Sample: Corylus avellana

Sequencing reads obtained from *C. avellana* mapped to the nuclear (54.8%), chloroplast (30.6%), and mitochondrial (14.6%) genomes. While all chloroplast-derived reads were successfully assigned to *C. avellana* (Figure 2B), mitochondrial reads were mapped to the *Betula pendula* genome, probably due to the lack of an available mitochondrial genome sequence for *C. avellana*.

**Figure 3:**
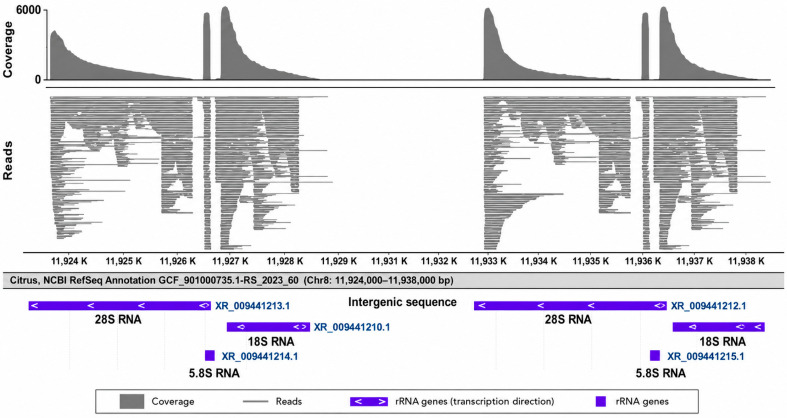
Sequence reads mapped to nuclear ribosomal gene cluster of *C. avellana* (assembly GCF_901000735.1).

In contrast to the chloroplast- and mitochondrial-derived reads, the majority of nuclear reads were assigned to *Alnus rubra*. To elucidate the cause of this discrepancy, sequence reads were mapped against the *C. avellana* reference genome (assembly GCF_901000735.1). Most reads mapped either to the chloroplast genome or chromosome 8, which contain duplicated rDNA clusters separated by a 5422-bp intergenic spacer (Figure 3).

**Figure 4:**
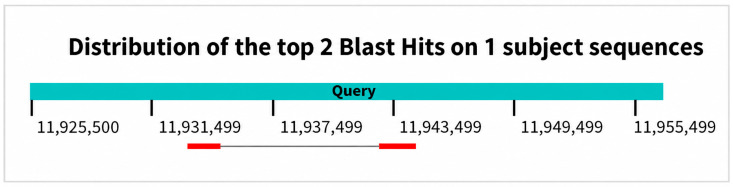
Example of BLASTn v.2.17.0 aligning of sequence read to *C. avellana* nuclear genome. Red line: positions of the 2 BLAST hits on the rDNA cluster sequence. The gray line between the red segments indicates the region spanned by the two hits on the rDNA cluster sequence.

Sequence alignment may misinterpret reads spanning adjacent rDNA clusters as single continuous sequences interrupted by divergent intervening regions. This can lead to inaccurate estimates of sequence similarity, resulting in erroneous taxonomic assignments (Figure 4), and is likely the reason why many reads were incorrectly assigned to *A. rubra*.

In addition to host-derived sequences, reads corresponding to the embryophyte environmental sample clone IMG124 (2.9%) and the fungal pathogen *Erysiphe corylacearum* (0.02%) were detected. The presence of *E. corylacearum* was confirmed by sequencing the ITS1–ITS2 region (GenBank accession no. PZ539357) and the RNA polymerase II second-largest subunit (*rpb2*) gene (GenBank accession no. PZ554488), indicating a latent infection of *C. avellana* leaves.

### 2.4. Animal Sample: Eisenia fetida

Most reads (83.6%) obtained from the *E. fetida* sample mapped to nuclear ribosomal genes. Additionally, 7.2% of the reads were assigned to the mitochondrial *cox1* gene, providing independent confirmation of species identity. The remaining fraction of reads consisted of low-abundance or unclassified sequences, reflecting the inherent error rate of nanopore sequencing and the limited taxonomic resolution of highly conserved rRNA regions, as well as incomplete representation of some taxa in reference databases. Beyond host-derived sequences, several bacterial taxa were detected, including *Verminephrobacter eiseniae* (1.4%), a known obligate nephridial symbiont of *E. fetida*, as well as *Acidovorax* sp. (1.2%), *Rhodococcus* sp. (1.2%), *Sphingomonas* sp. (1.1%), and *Variovorax paradoxus* (1.2%); and an uncultured bacterium accounted for 1.1% of the dataset. Low-abundance assignments to *Hexaplex trunculus* (1.0%) and *Urechis unicinctus* (1.0%) were also observed. These likely reflected conserved ribosomal regions or database-specific assignment artifacts rather than true biological presence. Indeed, while the percentages are comparable, the bacterial taxa are biologically plausible in this context, whereas the marine eukaryotes represent misassignments driven by highly conserved rRNA sequences.

Overall, the direct RNA sequencing datasets were dominated by ribosomal RNA reads, enabling reliable identification of target organisms across fungi, plants, and animals. Furthermore, the simultaneous detection of organellar transcripts and associated microbial taxa demonstrated the effectiveness of this approach in detecting both host identity and biological context within a single sequencing run.

## 3. Discussion

The ability to identify organisms without PCR amplification represents a conceptual challenge in molecular taxonomy. Traditional DNA barcoding approaches rely on the amplification of a limited set of standardized genetic loci using conserved primer pairs [30]. Although this strategy has been highly successful for species identification across many taxonomic groups, PCR-based methods can introduce biases related to primer mismatches, variable amplification efficiency, and marker selection [31,32]. Direct RNA sequencing offers an alternative framework by analyzing native RNA molecules without the need for targeted amplification [23,25,33]. Because this approach is not restricted to predefined genetic markers, it can simultaneously capture multiple sources of taxonomic information, including ribosomal RNA, organellar transcripts, and protein-coding genes [24,34]. The recovery of multiple transcription-derived markers within a single dataset may enhance resolution in species separation while decreasing reliance on single-marker barcodes. The current proof-of-concept study evidences the technical viability of the developed approach; however, further validation studies including a higher number of samples and replicates are necessary to assess its analytical robustness, repeatability, and reproducibility versus traditional primer-dependent sequencing techniques.

The approach developed in this study relies primarily on ribosomal RNA, which constitutes the majority of cellular RNA (80–90%) and consequently dominates direct RNA sequencing datasets [35,36]. Consistent with this expectation, most reads in our study corresponded to 18S and 28S rRNA, enabling reliable identification of target taxa across all analyzed organisms. However, the detection of ITS-derived reads was somewhat unexpected, given that the 18S, 5.8S, and 25S (or 28S in mammals) rRNAs are originally transcribed as a single precursor unit, known as 45S rDNA in plants, 47S rDNA in mammals, and 35S rDNA in yeasts. During the maturation of pre-rRNA, these transcribed spacers are sequentially eliminated through a series of endonucleolytic and exonucleolytic cleavages (reviewed in [37,38]).

Previous studies have successfully demonstrated the utility of ONT dRNA-seq in viral diagnostics. For instance, Tan et al. [39] showed that dRNA-seq enables rapid detection of porcine reproductive and respiratory syndrome virus. Similarly, Leigh et al. [40] evaluated the suitability of dRNA-seq for viral subtype identification, concluding that this method supports reliable subtype classification despite moderate error rates. Stenger et al. [41] further illustrated the power of ONT sequencing in rediscovering and genomically characterizing sowthistle yellow vein virus. Chalupowicz et al. [42] demonstrated that dRNA-seq enables the rapid identification of plant pathogens directly from infected tissues. Collectively, these studies showed that dRNA-seq can be used for virus identification.

Apart from viruses, dRNA-seq has rarely been used for species identification. A notable exception includes the works of Phannareth et al. [43] and Yang et al. [28], who successfully applied this technique to identify the plant-pathogenic bacterium *Ralstonia solanacearum* and food-borne bacteria, respectively. Furthermore, Semmouri et al. [29] demonstrated the effectiveness of dRNA-seq for metatranscriptomic analysis of marine zooplankton.

Compared with previous studies that have primarily focused on viral detection or transcriptomic profiling, our work extends the application of dRNA-seq to primer-independent species identification across multiple eukaryotic kingdoms. We demonstrated that naturally abundant ribosomal RNA provides a sufficient taxonomic signal to identify fungi, plants, and animals, while simultaneously revealing host-associated microbiota and co-occurring taxa. Consequently, this approach expands the utility of dRNA-seq from pathogen-focused diagnostics into a broader molecular taxonomy framework. Our results regarding *E. fetida* identification (Figure 2) aligned with those reported by Semmouri et al. [29].

Although the analysis primarily aimed to identify the host specimens, dRNA-seq additionally provided data on the associated microbiota. In the earthworm dataset, *Verminephrobacter eiseniae* was detected, in agreement with previous studies describing this bacterium as an obligate nephridial symbiont [44,45]. Similarly, detection of *Acidovorax* and other bacterial taxa reflects the microbiome associated with earthworm tissues [46,47]. In plant samples, the identification of *Erysiphe corylacearum* indicated latent fungal infection that would likely remain undetected using morphology-based diagnostics [48,49]. These findings suggested that dRNA-seq can simultaneously detect host identity and associated microbiota, providing a holistic view of biological samples.

A notable observation was the substantial contribution of organellar transcripts within the plant dataset. Approximately one-third of the reads mapped to chloroplast sequences, with additional reads originating from mitochondrial transcripts. Importantly, chloroplast-derived reads encompassed widely utilized plant barcoding markers, such as *rbcL*, demonstrating that dRNA-seq can recover standard taxonomic markers without targeted amplification. This multi-locus signal may improve confidence in species identification and reduce reliance on single-marker approaches. A comparative overview of sequencing approaches for species identification is presented in Table 1.

Furthermore, primer-free species identification by dRNA-seq offers a distinct economic advantage over multi-step HTS workflows (Table 2).

By eliminating the use of primers, polymerases, and amplification steps, our protocol enables significant cost reductions, with an estimated savings of 20–40% per sample. These economic benefits are particularly important for molecular taxonomy and diagnostics, where the use of quick, cost-efficient, and sustainable sequencing methods is increasingly essential.

However, several limitations must be considered. Genes encoding ribosomal RNA (rRNA) are organized in tandemly repeated clusters separated by intergenic spacers. Our results indicated that classification methods may misassign reads when adjacent rDNA units are interpreted as continuous sequences. Currently, to bypass this issue in plant species identification, the chloroplast genome can be used, as it offers a more stable and unambiguous resource for taxonomic assignment.

Another limitation of this method is that dRNA-seq requires high-quality starting RNA. While RNA used in this study had robust RIN values and did not impact the results of the study, the presence of more evident RNA degradation in samples with lower RIN scores could negatively influence adapter ligation and the downstream sequencing process. Therefore, further optimization of this workflow with lower-quality RNA is necessary.

Nanopore direct RNA sequencing has historically exhibited higher error rates than short-read technologies, potentially limiting the discrimination of closely related taxa with highly conserved nucleotide identity [39,40]. This is due to direct translocation of RNA strands through the pore, resulting in lower Q-scores compared to conventional cDNA sequencing, PacBio, or Illumina platforms. Thus, a high per-base error rate may mask the single-nucleotide polymorphisms or indel variants required for precise taxonomic assignment. The introduction of the RNA004 chemistry addresses these limitations through an optimized motor protein for faster translocation and a modified pore that measures 9-mer signals, resulting in higher basecalling accuracy [50,51]. Continuous advancements in chemistry and software are anticipated to improve accuracy and expand the technology’s application.

An additional constraint of the developed workflow is its reliance on high-quality RNA. Unlike traditional DNA sequencing, which can utilize archived and silica-dried specimens, dRNA-seq requires highly intact RNA and therefore demands caution in the collection and conservation of samples, particularly under field conditions.

Despite these challenges, dRNA-seq offers distinct advantages over traditional DNA-based barcoding. It couples taxonomic identification with transcriptional profiling, including organellar gene expression. In ecological contexts, such integrated taxonomic–functional assessments can provide deeper insights into organismal activity and community dynamics. The capacity to capture host identity, associated microbiota, and functional signals within a single workflow represents a substantial conceptual advancement. Consequently, primer-independent sequencing holds significant promise for biodiversity monitoring and bio-diagnostics. Because environmental samples typically comprise complex biological mixtures where universal primers exhibit amplification bias, an amplification-free approach yields a more representative overview of communities and facilitates the detection of rare or cryptic taxa. Furthermore, since RNA reflects active transcription, this method preferentially captures metabolically active community members, thereby effectively complementing DNA-based surveys that often amplify relic or extracellular DNA.

The present study was intended as a proof-of-concept study to assess whether direct RNA sequencing can provide sufficient taxonomic information for primer-less species identification across representative taxonomic units. Future studies should expand taxonomic sampling, increase the number of biological replicates, include independent operators and sequencing instruments, and develop specialized analytical tools optimized for long-read RNA classification. This will allow systematic benchmarking of dRNA-seq performance against established DNA barcoding methods in terms of diagnostic reproducibility, accuracy, and comparative performance through robust statistical analyses.

Consequently, at its current stage of development, the proposed dRNA-seq approach should be considered as complementary to existing DNA-based approaches, contributing to the development of future approaches. Ultimately, integrating direct RNA sequencing with complementary DNA-based approaches could yield robust and hybrid workflows for comprehensive biodiversity monitoring and molecular diagnostics.

In conclusion, our findings demonstrated that Oxford Nanopore Technology’s direct RNA sequencing establishes a proof of concept that enables primer-independent species identification across multiple eukaryotic kingdoms while simultaneously identifying host-associated microbiota and organellar transcription. By eliminating primer-induced amplification bias and moving beyond narrow, targeted pathogen detection, this innovative framework serves as a powerful addition to the molecular taxonomy toolkit. Driven by ongoing technical refinement and analytical advancements, direct RNA sequencing is well-positioned to evolve into a mainstream, unbiased strategy for rapid species identification across ecological, agricultural, and diagnostic applications. Although further validation using a larger number of samples and replicates, together with direct comparisons with conventional DNA barcoding methods, is necessary, the technical applicability of the developed amplification-free workflow demonstrated in this study opens perspectives for its use as a complementary approach in molecular taxonomy.

## 4. Materials and Methods

### 4.1. Sample Collection and RNA Isolation

Fungal, plant, and animal specimens were collected between July and September 2024. Sporocarps of *Tuber magnatum* and leaves of *Corylus avellana* were collected in the Turin area, Italy (July 2024). Specimens of the earthworm *Eisenia fetida* were collected in September 2024 at the Department of Agriculture and Waste Management, University of Rzeszów, Poland.

Total RNA was isolated using the RNeasy Mini Kit (Qiagen, Hilden, Germany) according to the manufacturer’s instructions. RNA purity and concentration were assessed using a NanoDrop 2000 spectrophotometer (Thermo Fisher Scientific, Waltham, MA, USA). RNA integrity was evaluated by electrophoresis on a 1.5% agarose gel stained with ethidium bromide. Only samples meeting quality and integrity criteria (RIN values ≥ 8) were selected as suitable for dRNA-seq and used for downstream analyses.

### 4.2. Poly(A) Tailing, Library Preparation, and Nanopore Direct RNA Sequencing

To enable direct RNA sequencing of non-polyadenylated transcripts, poly(A) tails were enzymatically added to total RNA [52]. Approximately 500 ng of native RNA per sample was treated with *E. coli* poly(A) polymerase (New England Biolabs, Ipswich, MA, USA). Reactions were incubated at 37 °C for 10 min using 5 U of polymerase. Following tailing, RNA was purified using RNAClean XP beads (Beckman Coulter, Brea, CA, USA) and eluted in 15 µL of nuclease-free water. Polyadenylated RNA was used for library preparation with the Oxford Nanopore Technologies Direct RNA Sequencing Kit (SQK-RNA002) following the manufacturer’s protocol (DRS_9195_v4_revF_11Dec2024). Sequencing libraries were loaded onto R9.4.1 flow cells (FLO-MIN106D) and sequenced on a MinION Mk1C device (Oxford Nanopore Technologies, Oxford, UK). Each sequencing run lasted approximately 6 h. Basecalling and run monitoring were performed using MinKNOW software (version 22.05.06).

### 4.3. Taxonomic Assignment and Data Analysis

Long-read nanopore datasets present unique challenges for taxonomic classification due to their characteristic error profiles and variable read lengths. Algorithms originally developed for short-read metagenomics may produce inflated false-positive assignments when applied directly to long-read data [53,54]. To address this issue, taxonomic classification in the present study employed the CCMetagen v1.2.3 pipeline, which implements the ConClave [55] sorting algorithm to reduce spurious matches across closely related reference sequences. This approach was effective for taxonomic assignments in complex metagenomic datasets [56] and outperformed several other methods (e.g., Kraken2, Centrifuge, and KrakenUniq) [57]. Reads were then compared against the full NCBI nucleotide database. Similarity thresholds for taxonomic assignment followed previously published guidelines: 98.4% for species-level identification, 96.3% for genus, 88.5% for family, 81.2% for order, 80.9% for class, and 50.0% for phylum-level assignments [58]. For selected analyses, reads were mapped to reference genomes using minimap2 [59] to investigate potential misclassification caused by ribosomal gene cluster organization.

Total genomic DNA was extracted from the same hazelnut leaf samples used for dRNA-seq species identification. DNA extraction using 5% Chelex (Bio-Rad, Hercules, CA, USA) was performed following the protocol by [60] using a NanoDrop 2000 spectrophotometer (Thermo Fisher Scientific, Waltham, MA, USA). The obtained DNA extracts were amplified by PCR in two genomic regions: the internal transcribed spacer (ITS) region [61,62] and the RNA polymerase second-largest subunit (*rpb2*) [63]. PCR products were purified using a QIAquick PCR Purification Kit (Qiagen, Hilden, Germany) following the manufacturer’s instructions, followed by sequencing in both directions at the BMR Genomics Centre (Padua, Italy).

## 5. Conclusions

This study demonstrated that Oxford Nanopore direct RNA sequencing (dRNA-seq) enables primer-independent species identification across plants, fungi, and animals. The results presented here confirmed the initial hypothesis that the natural abundance of ribosomal RNA and organellar transcripts was sufficient for accurate taxonomic assignment of the target species without reverse transcription or PCR amplification. Beyond identifying target host organisms, this methodology enabled simultaneous detection of associated bacteria, fungi, and insects, highlighting its high sensitivity and capacity to characterize entire biological assemblages from a single dataset.

In plant specimens, dRNA-seq recovered abundant chloroplast and mitochondrial transcripts—including loci commonly utilized in conventional DNA barcoding—thereby providing independent genomic validation of species identity. However, the complex organization of nuclear ribosomal gene clusters can confound automated classification, underscoring the need for refined bioinformatic workflows and highlighting the utility of chloroplast-derived sequence reads as a more stable alternative for precise plant species diagnostics.

By enabling taxonomic inference directly from native molecules, dRNA-seq significantly expands the molecular systematics landscape. Because highly abundant ribosomal transcripts contain both ultra-conserved and highly variable regions, they provide a robust phylogenetic signal across diverse kingdoms without targeted enrichment. This approach yields multiple informative loci within a single experiment, securing reliable identification while concurrently uncovering nested biological signals. This primer-free framework is particularly advantageous for taxonomic groups where conventional barcoding markers suffer from amplification bias or poor primer conservation. Anticipated advancements in nanopore chemistry and base-calling algorithms will drive down error rates and increase throughput, cementing RNA-based approaches in biodiversity research. Ultimately, as technical and analytical ecosystems mature, dRNA-seq is poised to become a mainstream strategy for rapid species identification and the holistic exploration of complex biological communities, while also offering substantial potential for real-time, on-site diagnostic applications.

## Figures and Tables

**Table 1:** Scientific comparison of major sequencing approaches relevant to identification, diagnosis, and biodiversity studies.

Approach	Starting Molecule and Library Principle	Typical Read Structure	Accuracy/Information Profile	Scientific Strengths	Scientific Limitations	Relevance for Identification, Diagnosis, and Biodiversity
Illumina DNA metabarcoding	DNA; PCR amplification of taxonomic markers such as 16S, ITS, 18S, or COI.	Short paired-end reads, commonly 2 × 150 or 2 × 300 bp.	Very high base-calling accuracy; strong marker-level quantification when reference databases are curated.	Robust, high-throughput, and widely benchmarked for community profiling.	PCR bias; limited species resolution when short markers are not discriminant; no direct evidence of transcriptional activity.	Excellent for routine biodiversity surveys and taxonomic screening, but biologically limited for active-process inference.
Illumina RNA-seq	RNA converted to cDNA; fragmentation, adapter ligation, and, usually, PCR amplification.	Short reads, usually 75–150 bp.	Very high base-calling accuracy; strong quantitative power for differential expression.	Mature analytical ecosystem; excellent for gene-expression quantification.	Native RNA signal is lost after cDNA conversion; short reads may require transcript reconstruction; RT/PCR bias may occur.	Strong for transcript abundance studies, but less suitable for native RNA features or direct long-read taxonomic assignment.
Oxford Nanopore DNA ligation sequencing	DNA; end-preparation and adapter ligation, e.g., SQK-LSK114.	Long reads from kilobases to ultra-long molecules, depending on DNA quality.	Current Kit 14 chemistry supports high nanopore accuracy and long-read continuity.	Long-read contiguity; real-time sequencing; useful for genome assembly, structural variants, long amplicons, and metagenomics.	Requires good DNA quality; no direct information on RNA activity.	Highly useful for long-amplicon identification, metagenomics, and genome-level characterization.
Oxford Nanopore rapid DNA barcoding	DNA or amplicons; rapid transposase-based barcoding, e.g., SQK-RBK114.24 or SQK-RBK114.96.	Long reads, often shorter and more variable than ligation libraries.	Comparable to current ONT Kit 14 chemistry, depending on basecalling and sample quality.	Fast, simple, and suitable for multiplexed diagnostic workflows.	Less control over insert size than ligation libraries; DNA-based signal only.	Attractive for rapid DNA-based identification and screening, including small-scale runs.
PacBio HiFi DNA sequencing	DNA; circular consensus sequencing.	Long, highly accurate reads, commonly 10–25 kb.	Very high consensus accuracy, often approaching short-read accuracy while retaining long reads.	Excellent for high-quality genome assembly, haplotyping, and structural variant analysis.	Higher cost and generally less flexible for low-throughput diagnostic use.	Powerful for high-resolution genome characterization; often excessive for routine biodiversity screening.
PacBio Iso-Seq	RNA converted to full-length cDNA; full-length transcript sequencing.	Long, full-length, transcript-derived reads.	High accuracy when generated as HiFi reads.	Excellent for isoform discovery, transcript annotation, and alternative splicing analysis.	RNA is converted to cDNA; native RNA modifications and direct RNA signal are not retained.	Highly suitable for transcript annotation, but not a native RNA approach.
Oxford Nanopore cDNA sequencing	RNA converted to cDNA; nanopore sequencing of cDNA, with or without PCR, depending on protocol.	Long transcript-derived reads.	Good transcript-level information, dependent on RT, amplification, and basecalling quality.	Captures longer transcript structures than short-read RNA-seq; useful when RNA quantity or integrity limits direct RNA sequencing.	RT bias; PCR bias when amplified; native RNA modifications are lost.	Useful for long-read transcriptomics, but biologically less direct than native RNA sequencing.
Oxford Nanopore direct native RNA sequencing	Native RNA; RNA molecules are sequenced directly. Reverse transcription is used only to synthesize a stabilizing cDNA strand, while RNA is the sequenced molecule.	Native RNA long reads, with length depending strongly on RNA integrity.	Lower base-level accuracy than Illumina and PacBio HiFi, but improved with RNA004 chemistry and modern basecalling; retains native RNA signal.	PCR-free; avoids full conversion of the biological signal into cDNA; preserves strand-specific native RNA information; can support transcript-level identification and potential RNA-modification analysis; targets transcriptionally active organisms.	Requires high-quality RNA; lower throughput than Illumina; poly(A)+ enrichment or targeted enrichment may be needed; more specialized bioinformatics.	Particularly attractive for integrated identification, diagnosis, and biodiversity studies because it links taxonomic detection with native RNA/transcriptional activity while minimizing amplification-related bias.

The highlighted row indicates the method with the strongest conceptual fit for PCR-free native RNA-based identification, diagnosis, and biodiversity studies.

**Table 2:** Economic and operational comparison of major DNA- and RNA-sequencing approaches.

Approach	Representative Platform/Chemistry	Relative Cost Per Project	Cost Drivers	Hands-On Time and Workflow Complexity	Scalability/Throughput	Operational Advantages	Operational Constraints
Illumina DNA metabarcoding	MiSeq/NextSeq short-read amplicon sequencing.	Low to medium, especially with high sample multiplexing.	Primer design, PCR, indexing, sequencing depth, database curation, and bioinformatics.	Moderate; PCR and indexing are routine but require contamination control.	Very high; efficient for hundreds of samples.	Cost-effective for large biodiversity surveys; standardized wet-lab and bioinformatic pipelines.	Best suited to short-marker questions; service provider turnaround may limit rapid decisions.
Illumina RNA-seq	Stranded mRNA-seq or total RNA-seq.	Medium to high, depending on rRNA depletion, depth, and replication.	RNA quality control, rRNA depletion or poly(A)+ selection, library prep, sequencing depth, and differential expression analysis.	Moderate to high; RNA handling and library prep are more demanding than DNA metabarcoding.	High; strong for replicated expression experiments.	Economically efficient when many samples require quantitative expression profiling.	Less flexible for rapid small runs; native RNA information is lost.
Oxford Nanopore DNA ligation sequencing	SQK-LSK114 with FLO-MIN114/FLO-PRO114.	Medium; cost-efficient when long-read information is required.	Flow cell, ligation kit, DNA extraction quality, cleanup reagents, and desired depth.	Moderate; more steps than rapid protocols but good control over library quality.	Medium to high, depending on flow cell and barcoding strategy.	Portable, real-time sequencing; strong value when long contiguity is important.	Requires high-quality DNA and careful size preservation; not optimized for fastest sample-to-answer workflow.
Oxford Nanopore rapid DNA barcoding	SQK-RBK114.24/SQK-RBK114.96 with FLO-MIN114 or FLO-FLG114.	Low to medium; attractive for small batches and multiplexed diagnostics.	Rapid barcoding kit, flow cell or Flongle, DNA input quality, and number of samples per run.	Low; among the simplest ONT DNA workflows.	Medium; 24- or 96-barcode configurations support multiplexing.	Rapid preparation; compatible with field-oriented and small-laboratory workflows.	Lower control over fragment size; DNA-only information; output depends on flow cell choice.
PacBio HiFi DNA sequencing	Revio/Sequel IIe HiFi sequencing.	High; often most economical through core facilities or service providers.	Instrument access, library size selection, SMRT cells, sequencing depth, and sample QC.	High at library-prep level, usually handled by specialized facilities.	High for genome projects, less flexible for small diagnostic batches.	Excellent data quality for assemblies and variant discovery.	Cost and logistics can be limiting for rapid or low-throughput diagnostic applications.
PacBio Iso-Seq	PacBio Iso-Seq full-length cDNA transcript sequencing.	High.	High-quality RNA, cDNA synthesis, size selection, SMRT sequencing, and specialized analysis.	High; requires careful RNA and full-length cDNA preparation.	Medium; best for selected samples rather than very large routine screens.	High-value data for transcript annotation and isoform discovery.	Less economical for routine biodiversity or diagnostic screening; not native RNA.
Oxford Nanopore cDNA sequencing	ONT direct cDNA or PCR-cDNA workflows.	Medium.	RNA extraction, cDNA synthesis, optional PCR, nanopore kit, flow cell, and analysis.	Moderate; more tolerant than direct RNA but includes RT and sometimes PCR.	Medium; barcoding options can improve scalability.	Useful compromise when long transcript reads are needed but native RNA sequencing is not feasible.	RT/PCR steps add bias and cost; native RNA modifications are not retained.
Oxford Nanopore direct native RNA sequencing	SQK-RNA004 with RNA flow cells FLO-MIN004RA/FLO-PRO004RA.	Medium to high per run, but scientifically efficient when native RNA information is required.	RNA-specific flow cell, SQK-RNA004 kit, high-quality RNA extraction, poly(A)+ or targeted enrichment, QC, and specialized analysis.	Moderate to high; RNA integrity and handling are critical.	Lower than Illumina for very large sample sets; suitable for focused high-information experiments.	High information density: PCR-free native RNA sequencing can provide taxonomic signal, transcript-level information, and potential RNA-modification signal in a single conceptual workflow.	Requires excellent RNA quality and careful experimental design; per-sample cost can be higher when few samples are loaded.

Note. Relative cost categories are intended for manuscript-level comparison and should be updated with local quotations before procurement. The highlighted row indicates the method with the strongest conceptual fit for PCR-free native RNA-based identification, diagnosis, and biodiversity studies.

## Data Availability

The data presented in this study are openly available at NCBI at https://www.ncbi.nlm.nih.gov/nuccore/PZ539357.1/, accessed 22 June 2026, reference numbers PZ539357 and PZ554488. Sequences of ITS and *rpb2* genes are deposited at NCBI (GenBank accession nos. PZ539357 and PZ554488, respectively). Other data are available from the authors upon request.
